# Decreased serum PON1 arylesterase activity in familial hypercholesterolemia patients with a mutated *LDLR* gene

**DOI:** 10.1590/1678-4685-GMB-2016-0287

**Published:** 2018-07-23

**Authors:** Muhammad Idrees, Abdul Rauf Siddiq, Muhammad Ajmal, Muhammad Akram, Rana Rehan Khalid, Alamdar Hussain, Raheel Qamar, Habib Bokhari

**Affiliations:** 1 COMSAT Institute of Information Tecnology, Islamabad, Pakistan

**Keywords:** Paraoxonase-1, hypercholesteremia, arylesterase, LDLR mutation

## Abstract

Paraoxonase 1 (PON1) is a serum enzyme associated with high density lipoprotein (HDL) regulation through its paraoxonase and arylesterase activity. PON1 inhibits the oxidation of HDL and low density lipoprotein (LDL), and is involved in the pathogenesis of a variety of diseases including atherosclerosis. Conversely, mutations in the low density lipoprotein receptor (*LDLR*) result in failure of receptor mediated endocytosis of LDL leading to its elevated plasma levels and onset of familial hypercholesterolemia (FH). In the current study we investigated the role of PON1 polymorphisms rs662; c.575A > G (p.Gln192Arg) and rs854560; c.163T > A (p.Leu55Met) in a large family having FH patients harboring a functional mutation in LDLR. Genotypes were revealed by RFLP, followed by confirmation through Sanger sequencing. PON1 activity was measure by spectrophotometry. Our results show significantly reduced serum paraoxonase and arylesterase activities in FH patients compared with the healthy individuals of the family (p < 0.05). PON1 QQ192 genotype showed a significantly higher association with FH (p=0.0002). PON1 Q192 isoform was associated with reduced serum paraoxonase activity by *in silico* analysis and PON1 R192 exhibited higher serum paraoxonase and arylesterase activity than the other polymorphs. Our results highlight that the combination of *LDLR* mutations and PON1 MMQQ genotypes may lead to severe cardiac events.

## Introduction

The human paraoxonase (PON) gene family located on the long arm of chromosome 17 consists of three members, each of which coding for three different calcium dependent esterases: PON1, PON2, and PON3 (La Du *et al.*, 1993; Li *et al.*, 2003; Mackness and Mackness, 2015). PON1 and PON3 are plasma HDL-associated enzymes with antioxidant activities, albeit with differences (Mackness *et al.*, 1993). PON1 serum concentration is affected by inflammation and serum levels of oxidized-LDL (Ceron *et al.*, 2014; Vakili *et al.*, 2014; Mackness and Mackness, 2015). PON3 on the other hand is far less expressed and is not influenced by inflammation or oxidized lipids (Reddy *et al.*, 2001). PON2 is an intracellular enzyme with ubiquitous expression and is thought to protect against oxidative stress (Ng *et al.*, 2001).

In the current study we investigated PON1 in familial hypercholesteremia due to its role in diverse physiological and pathophysiological functions, including atherosclerosis and inflammatory diseases (La Du, 1996; Li *et al.*, 2003). PON1 is associated with high density lipoprotein (HDL) in human serum and prevents oxidation of both low density lipoprotein (LDL) and HDL (Aviram *et al.*, 1998; Durrington *et al.*, 2001; Mackness *et al.*, 1996). The inhibition of LDL and HDL oxidation may protect against various pathologies including cardiovascular diseases (CVD); PON1, therefore, is also considered the gene of longevity (Lescai *et al.*, 2009, Martinelli *et al.*, 2013). A relationship between paraoxonase 1 (*PON1*) genotype status, anti-oxidant, and anti-atherogenic capacity of the enzyme has been suggested previously (Mackness *et al.*, 1993, 2002; Rosenblat *et al.*, 2006). In addition, PON1 arylesterase/paraoxonase activities have been shown to be inversely correlated to the risk of coronary heart diseases and hypercholesterolemia (Humbert *et al.*, 1993; Garin *et al.*, 1997; Bryk *et al.*, 2005).

Functional mutations in LDLR gene cause the monogenic form of familial hypercholesterolemia (FH) (Diakou *et al.*, 2011; Ahmed *et al.*, 2012). Recently, it was also shown that increased serum paraoxonase activity in *LDLR* (-/-) mice significantly inhibits progression of atherosclerosis (Leckey *et al.*, 2010). However, the role of PON1 activity has not been studied previously in individuals with mutated *LDLR*. A large Pakistani family with *LDLR* associated FH was investigated in this study to understand the role of PON1 in the protection against atherosclerosis (Ajmal *et al.*, 2010). In this study, we report on the role of *PON1* coding sequences of single nucleotide polymorphisms (SNPs) rs662 (c.575A > G; p.Gln192Arg) and rs854560 (c.163T > A (p.Leu55Met) in relation to resultant paraoxonase and arylesterase activity in hypercholesterolemia patients with mutated *LDLR*. The structural and functional aspects of these SNPs have also been studied to explore how different allozymes affect and mediate the paraoxonase and arylesterase activities of the enzyme.

## Subjects and Methods

### Subjects

A large consanguineous Pakistani family (n=34) with *LDLR* mutation (c. 2416_2417InsG) presenting clinical FH was identified, including 10 patients suffering from FH and 24 healthy individuals (Ajmal *et al.*, 2010). All subjects were screened for the presence/absence of CHD, diabetes, hypertension, malignant tumors, and acute or chronic infectious diseases. The study was approved by the Ethics Committee and Institutional Review Board of the Department of Biosciences, COMSATS Institute of Information Technology, Islamabad, Pakistan. All patients and participating healthy members of the family were informed about the study in their local language and written consent was obtained from them prior to inclusion in the study.

### Samples

Blood (5 mL) was drawn after 12–14 h fasting and collected in separate tubes for DNA extraction by organic method (Helms *et al.*, 1985) and serum separation for the determination of lipid profile and enzyme activities. For DNA extraction, blood was collected in acid citrate dextrose (ACD) vacutainer (Becton–Dickinson, Franklin Lakes, NJ) and for serum separation, blood was collected in Z Serum Sep Clot Activator vacutainer tubes (Greiner bio-one, Munich, Germany). Serum was separated from clotted blood by centrifuging the vacutainers at 3000 rpm for 10 min, at 4 °C.

### Determination of PON1 SNPs

Genomic DNA was extracted from peripheral blood leukocytes using standard procedures (Helms *et al.*, 1985). Two sets of primers were used for genotyping the polymorphisms of codon 192 and codon 55 in the *PON1* gene as described by Motti *et al.* (2001). The primer sequences for *PON1* Q192R (rs662) and *PON1* L55M (rs854560) were as follows: *PON1* Q192R-forward primer TTG AAT GAT ATT GTT GCT GTG GGA CCT GAG and *PON1* Q192R-reverse primer CGA CCA CGC TAA ACC CAA ATA CAT CTC CCA G*a*A; *PON1* L55M -forward primer GAG TGATGT ATA GCC CCA GTT TC and *PON1* L55M-reverse primer AGT CCATTA GGC AGTATC TCC *g*. The reverse primers contained mismatched nucleotides as indicated by lower case letters in italics. This allowed a restriction site for *Hinf*I (G/ANTC) to be introduced into the DNA amplification products in the presence of the polymorphisms arginine-*PON1*-192 or leucine-*PON1*-55. *PON1* gene segments were amplified by PCR using 0.3 mM deoxyribonucleotide triphosphates (dNTPs), 1x PCR buffer (10 mM Tris–HCl pH 9.0, 50 mM KCl), 2.0 mM MgCl_2_, 0.5 mM of each primer (forward and reverse), 1.5 U of *Taq* Polymerase, and 50 ng of genomic DNA. The thermal cycling consisted of an initial denaturation at 95 °C for 4 min, followed by 35 cycles of amplification consisting of denaturing at 95 °C for 45 s, primer annealing at 55 °C for 1 min and chain extension at 72 °C for 1 min. A final extension step was performed at 72 °C for 10 min.

### Screening of the amplified products

PCR products were purified using a DNA extraction kit (Fermentas Life Sciences, Burlington, Ontario, Canada) and subjected to restriction with H*inf*1 enzyme to screen for the type of SNP in the target sequence. Digested fragments were resolved on 8% polyacrylamide gel to reveal the genotypes (Figure S1). The results were analyzed to compare the prevalence of *PON1* L55M and *PON1* Q192R SNPs. To confirm the DNA sequence, amplified products were also subjected to bidirectional sequencing by the Sanger sequencing method to reveal the genotypes of all the individuals.

### Measurement of PON1 paraoxanase activity

Paraoxonase activity was determined by measuring the increase in absorbance at 412 nm (Thermo Scientific GENESYS 10 UV Scanning UV/Visible Spectrophotometer) due to the formation of 4-nitrophenol (using paraoxon as substrate) (Zehra *et al.*, 2009). The assay mixture contained 1.0 mM paraoxon and 1.0 mM CaCl_2_ in 50 mM glycine/NaOH buffer (pH 10.5). The amount of 4-nitrophenol liberated was calculated from the molar coefficient 18,290/Mcm.

### Measurement of PON arylesterase activity

Arylesterase activity was determined using an assay mixture containing 1.0 mM phenylacetate and 0.9 mM CaCl_2_ in 20 mM Tris-HCl buffer (pH 8.0). The rate of hydrolysis was monitored at 270 nm. The results were calculated using extinction coefficient 1310/Mcm.

### Other paramenters

The lipid profile, including total cholesterol (TC), triglycerides (TG), LDL-cholesterol (LDL-C) and HDL cholesterol (HDL-C), of all the subjects was obtained using a Roche/Hitachi automated system with commercial kits for CHOL (Cholesterol CHOD-PAP), LDL-C plus 2nd generation (LDL Cholesterol), HDL-C plus 3rd generation (HDL-Cholesterol) and TG (Triglyceride GPO-PAP) (all from Roche Diagnostics, Germany).

### Statistical analysis

Results for continuous variables are reported as mean ± SD. Differences among patients and healthy family members were assessed using Chi-square (χ^2^) and Fisher’s Exact test. *P*-values less than 0.05 were considered statistically significant. Continuous variables were analyzed using ANOVA whereas categorical variables were compared by Chi-squared and Fisher’s Exact test.

### Modeling the structure of PON allozymes

Structures of the four different PON1 allozymes, L55, M55, Q192 and R192, were modeled at Modeller (Eswar *et al.*, 2006) upon an already determined structure of human PON1 at 2.2 Å resolution with an R-factor 0.217 (Harel *et al.*, 2004a,b). All four models were energy minimized and validated for various quality factors, which included Ramachandran plot for the backbone dihedral ψ and φ, bad angles, bad bonds, steric clashes, and Z score of the model. 

### Assessing paraoxonase and arylesterase activities *in silico*

To assess the paraoxonase and arylesterase activities of PON1 allozymes Q192 and R192, *in silico* molecular docking was done at Autodock, developed against Paraoxon and Phenyl Acetate and retrieved from Zinc database (Irwin and Shoichet, 2005; Morris *et al.*, 2009). The docked conformations of the ligands were ranked on the basis of binding affinity, analyzed, and assessed at Lig Plot+ and UCSF Chimera (Pettersen *et al.*, 2004; Laskowski and Swindells, 2011). The allozymes L55 and M55 were also analyzed to explore why PON1 L55 has been reported to show high seral concentration than M55 polymorph (Bryk *et al.*, 2005).

## Results

**Figure 1:**
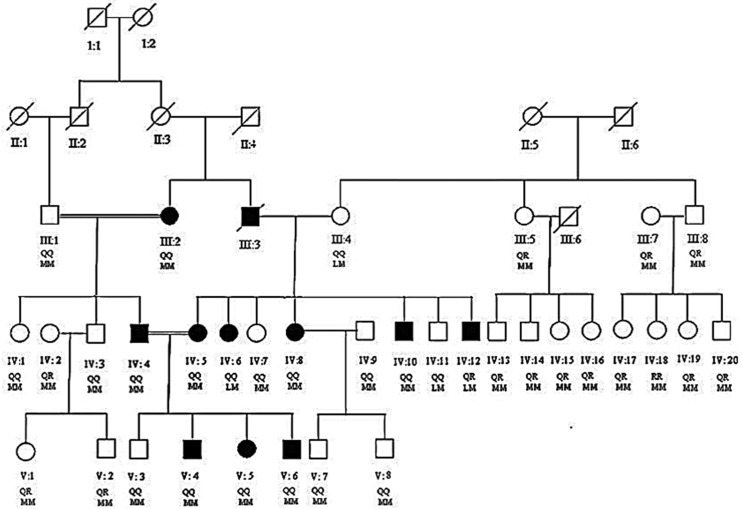
Pedigree of hypercholesterolemia family. Filled boxes and circles represent male and female patients respectively. Empty boxes and circles represent male and female carriers respectively. Modified from Ajmal *et al.* (2010).

All the living individuals of the family (Figure 1) were sampled and their *PON1* genotypes and phenotypes were determined. Demographic characteristics, lipid profiles, and /insparaoxonase and arylesterase activities in hypercholesterolemia patients and healthy individuals are summarized in Table 1.

**Table 1:** Blood chemistry and clinical data of the affected and normal individuals of the studied family.

Characteristics	Patients (n = 10)	Control (n = 24)	P-value
Age (Years)	22.5 ± 16.8	26.5 ± 14.6	NS
Male: Female	5:5	12:12	1
BMI (kg/m^2^)	20 ± 3.2	21 ± 3.6	NS
TC (mg/dL)	422.2 ± 181.5	184.9 ± 32.5	< 0.0001
TG (mg/dL)	167.6 ± 67.1	135.7 ± 80.1	0.28
LDL-C (mg/dL)	323.6 ± 149.6	108.5 ± 26.8	< 0.0001
HDL-C (mg/dL)	38 ± 8.4	45.0 ± 11.0	0.084
Paraoxonase activity (U/L)	116.5 ± 40.3	172.5 ± 61.6	0.001
Arylesterase activity (kU/L)	168.3 ± 24.8	210.7 ± 37.9	0.002
Xanthomas	1 (10%)	0 (0%)	—
CHD	1 (10%)	0 (0%)	—

Values are given as means ± SD (*p* < 0.05), continuous variables were analyzed using ANOVA whereas categorical variables were compared by Chi-square. NS: not statistically significant; BMI: body mass index; TC: serum total cholesterol; TG: serum total triglyceride; HDL-C: high-density lipoprotein cholesterol; LDL-C: low-density lipoprotein cholesterol; CHD: coronary heart disease risk.

### Clinical data

There was no significant difference between patients and healthy individuals with respect to mean ± SD values of age, gender and body mass index (BMI). Routine laboratory findings of lipid profile indicated significant differences between the patients and healthy individuals for TC (*p* < 0.0001) and LDL-C (*p* < 0.0001) while differences between the TG (*p* = 0.28) and HDL-C (*p* = 0.084) were not significant. Paraoxonase (*p* = 0.001) and arylesterase activities (*p* = 0.002) were significantly lower in patients compared to healthy individuals of the family.

### PON1 polymporphisms genotyping data

PCR amplification results and restriction fragment length polymorphism (RFLP) results of amplified products were in accordance with the specific band sizes reported previously (Motti *et al.*, 2001). Sequencing results confirmed the PCR-RFLP based identification of SNPs. The prevalence of various genotypes (*PON1* SNPs and combinations) is given in Table 2. *PON1* M55 and Q192 allele prevalence was highly correlated with individuals with FH. Similarly, 80% of patients showed M55M55 genotype whereas 90% showed Q192Q192 genotype. *PON1* L55 and R192 alleles were deficient in patients compared to healthy individuals of the family. Conversely, *PON1* M55 and Q192 alleles were found significantly higher in patients. Similarly, the haplotype MM/QQ was prevalent in 80% of the FH patients whereas 10% of the FH patients exhibited LM/QQ and LM/QR haplotypes.

**Table 2:** Paraoxonase-1 allele, genotype, and haplotype distributions of L55M and Q192R polymorphisms in patients and healthy individuals of the hypercholesterolemia family.

	Characteristics (Allele/Genotypes)	Patients (n = 10)	Controls (n = 24)	*p*
Alleles	L55	2 (10%)	2 (4.2%)	0.336
	M55	18 (90%)	46 (95.8%)	0.927
	Q192	19 (95%)	33 (68.7%)	**0.0164**
	R192	1 (5%)	15 (62%)	0.998
Genotypes	L55L55	0 (0%)	0 (0%)	1
	L55M55	2 (20%)	2 (8.3%)	0.334
	M55M55	8 (80%)	22 (91.7%)	0.933
	Q192Q192	9 (90%)	10 (41.7%)	**0.0113**
	Q192R192	1 (10%)	13 (54.2%)	0.999
	R192R192	0 (0%)	1 (4.2%)	1
Haplotypes	M55M55/Q192Q192	8 (80%)	8 (33.3%)	**0.017**
	L55M55/Q192Q192	1 (10%)	2 (8.3%)	0.661
	L55M55/Q192R192	1 (10%)	0 (0%)	0.294
	M55M55/Q192R192	0 (0%)	13 (54.2%)	1
	M55M55/R192R192	0 (0%)	1 (4.2%)	1

*p* < 0.05 was considered statistically significant; data were analyzed using Fisher’s Exact Test.

### Paraoxonase and arylesterase activities *in silico*

Both paraoxon and phenyl acetate were docked against allozymes of the PON1, and showed a higher binding affinity to PON1 R192 than Q192. The binding affinity of paraoxon to PON1 Q192 was observed to be -5.6 kcal/mol whereas for PON1 R192 the biding affinity has been significantly higher, i.e., 6.1 kcal/mol. In case of phenyl acetate (PA), the biding affinity for the allozyme R192 was observed to be 6.3 kcal/mol; however, it was 5.7 kcal/mol for Q192 (Table 3).

**Table 3:** Binding affinities of PON1 Q192 and R192 against paraoxon and phenyl acetate.

PON1Allozyme	Ligand	Binding Energy (kcal/mol)
R192	Paraoxon	-6.10
	Phenyl acetate	-6.30
Q192	Paraoxon	-5.60
	Phenyl acetate	-6.00

The binding pocket of PON1 is perfectly designed to spatially accommodate a lactone or arylester, with its distal end distributed by highly basic residues, N168 and N224, to interact with NO_2_ of paraoxon or OH of phenylacetate through hydrogen bonds. This arrangement helps positioning the acyl bond in proximity to the catalytic dyad of histidine residues H115 and H134, which has been reported to deprotonate a water molecule to generate a hydroxyl ion, thereby hydroxylating the lactones (paraoxon) and arylesters (phenylacetate) (Khersonsky and Tawfik, 2006; Ben-David *et al.*, 2012).

**Figure 2:**
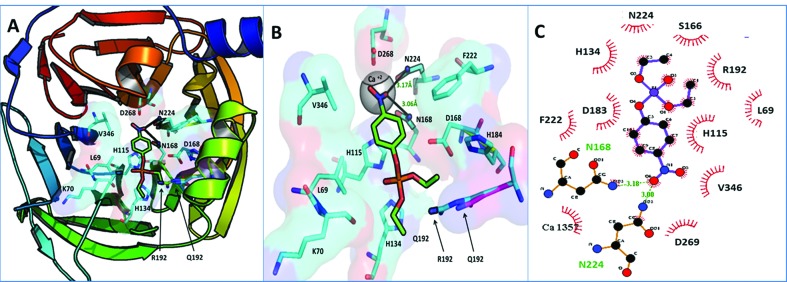
Docked conformations of paraoxon in PON1 binding pocket. (A) Paraoxon bound in PON1 binding pocket. (B) Zoomed in binding pocket of PON1, paraoxon terminally making two hydrogen bonds with N168 and N224. Notice that R192 adopts a conformation more proximal to the binding pocket opening than that of Q192, steering the ligand to adopt the most suitable conformation for paraoxonase activity by H 115 and H134 dyad. (C) 2D illustration of paraoxon binding in PON1 binding pocket; paraoxon forms two hydrogen bonds with Asn 224 (3.00 Å) and Asn 168 (3.18 Å) shown in green while a number of hydrophobic interactions with the residues configure the binding pocket.

Our results suggest that position 192 lies near the inner lining of the PON1 binding pocket opening. This position is critical because of its sidechain lying closer to the oxygen atoms of acyl group, which is a positively charged basic residue and has been selected by PON1 fold for this position over the course of time. A long sidechain of R192 with highly positively charged terminalguanidinium (HNC(NH_2_)_2_) mediates with acyl oxygen atoms then a small sized lysine sidechain with just one –NH_3_^+^, R192 thus helps in positioning the acyl bond of the substrate to the catalytic dyad of H115 and H134, thus the higher arylesterase and paraoxonase activity of R192 allozyme (Figure 2).

## Discussion

This is the first study of PON1 activity and region polymorphisms coding in a family with FH with *LDLR* mutation, although in a couple of recent studies, the role of rs662 was assessed in relation to serum lipid levels and coronary artery disease (Liang, 2016; Chen *et al.*, 2017). *PON1* genotype and activity are not being monitored in routine clinical practice for the management of hypercholesterolemia. Understanding a possibly protective role of PON1 activity in patients susceptible to atherosclerosis can help in taking early preventive measures for improvement of life span of hypercholesterolemia patients.

The contribution of PON1 in CVD is minor in healthy populations but it is known that genotypes with minor effects in general population may have more pronounced effects in patients, for example in FH cases (Leus *et al.*, 2001; Wiegman *et al.*, 2004). The beneficial effects of PON1 on the inhibition of atherosclerosis might be more pronounced in FH patients because they are more prone to develop atherosclerosis than the general population (van Himbergen *et al.*, 2005).

Low PON1 activity has been reported in previous studies as one of the leading factors causing atherosclerosis and myocardial infarction (Ayub *et al.*, 1999; James *et al.*, 2000; Mackness *et al.*, 2002; van Himbergen *et al.*, 2005; Bryk *et al.*, 2005; Gur *et al.*, 2006). Numerous clinical studies have shown an association of low PON1 activity with atherosclerosis and cardiovascular diseases (Jarvik *et al.*, 2000; Mackness *et al.*, 2003; Gur *et al.*, 2006; Rosenblat *et al.*, 2006; Soran *et al.*, 2008; Sun *et al.*, 2016; Verit *et al.*, 2008). Thus, significantly decreased paraoxonase and arylesterase activities in patients compared to the healthy individuals in the present study, indicate an increased risk of atherosclerosis in patients. One of the patients (IV-4) in the current family had a history of CVD in addition to FH. He had premature coronary artery disease and had suffered from myocardial infarction at an early age, which may have been due to decreased paraoxonase and arylesterase activity. The lipid profile of this 37-year-old male was not considerably elevated compared to other patients in this family.

Patient V-6 was identified with xanthomas in addition to FH, but without any history of CVD. However, his levels of TC (917 mg/dL) and LDLC (728 mg/dL) were markedly high and HDL-C (22 mg/dL) was lower compared to other FH patients in the family. The presence of tendon xanthomas is high risk factor of CVD among patients with FH, which along with decreased paraoxonase and arylesterase activities may lead to atherosclerosis (Jarvik *et al.*, 2000; Mackness *et al.*, 2003; Gur *et al.*, 2006; Rosenblat *et al.*, 2006; Soran *et al.*, 2008; Sun *et al.*, 2016; Verit *et al.*, 2008).

The low levels of serum paraoxonase and arylesterase activities of patients in this study may be due to their genetic makeup (*PON1* coding region SNPs). The L55M polymorphism affects the enzyme concentration (plasma PON1 protein levels indicated by serum arylesterase activity), whereas the Q192R polymorphism affects the catalytic efficiency (serum paraoxonase activity), but not the concentration (Bryk *et al.*, 2005). The PON1 M55 is associated with low plasma PON1 level (Blatter *et al.*, 1993; Mackness *et al.*, 1998a,b). The PON1 R192 allozyme hydrolyzes paraoxon more readily than Q192 (Costa *et al.*, 2005). In our study, the high prevalence of M55 allele in the patients may have been responsible for the low levels of PON1 activity (Aviram *et al.*, 2000). The frequency of the low-activity allele (Q192) and unstable form (M55 isoform, which is sensitive to proteolysis) was collectively higher in patients of this family indicated by high prevalence of MMQQ genotype (8, 80%), which may significantly reduce the function of PON1 for protection against atherosclerosis. The generally low frequency of the M55 is such that in some studies the MM genotype is not observed at all (Santos *et al.*, 2005). M is not favored in human populations, but it is kept in populations in heterozygous individuals. The L55M mutation may considerably affect PON1’s stability and thereby account for the lower enzymatic activity because M55 isoform is an unstable form (sensitive to proteolysis) (Harel *et al.*, 2004a,b; Mackness *et al.*, 1998a).

Indeed, PON1 phenotype (paraoxonase and arylesterase activities) has been shown previously to be a better predictor of vascular disease than PON1 Q192R or PON1 L55M genotypes due to possible effects of other genetic and non-genetic factors (Costa *et al.*, 2005; Jarvik *et al.*, 2000; Rosenblat *et al.*, 2011). Therefore, upregulation of PON1 levels by non-genetic factors (Rosenblat *et al.*, 2011) can have a potential advantage in such cases for better and achievable protection against atherosclerosis. Thus, our results combined with previously published data indicate the need of regular monitoring and upregulation of serum paraoxonase activity in patients with hypercholesterolemia for prevention of atherosclerosis. Our results highlight the importance of exploring PON1 as a therapeutic agent to accommodate the lower level of plasma PON1 in patients susceptible to atherosclerosis.

In the current study, the *LDLR* mutated patients had low level of PON1, which may indicate some gene-regulation action of *LDLR* and *PON1*, thus needing further investigations. With the current data, it is difficult to speculate how *LDLR* and *PON1* are involved in regulating each other.

Both the hypercholesterolemia and PON1 deficiency are independent risk factors for the development of atherosclerosis. In addition to controlling high levels of cholesterol, there is a need to regularly monitor PON1 status of hypercholesterolemia patients and normal family members. A better understanding of factors upregulating PON1 status in humans will have a significant public health impact by saving patients who are otherwise susceptible to atherosclerosis due to their deficient PON1 status. In the current study, controls from the general population were not screened, and this is a limitation of the study. However, based on the current findings, future studies can be designed to screen the population for PON1 and further investigate its role in cardiovascular diseases.

## Conclusion

This study aimed at exploring the implications of PON1 polymorphism in the individuals affected by familial hypercholesterolemia (FH). The role of PON1 and its various polymorphs has not been studied previously in FH subjects. This work sought to investigate paraoxonase and arylesterase activity of various PON1 SNPs in individuals with mutated *LDLR*, thereby explore their role in the development of FH. The results suggest that most of the hypercholesterolemia patients with *LDLR* mutation have homozygous M55 and Q192 *PON1* genotype, thus combination of MMQQ *PON1* genotype and *LDLR* mutation might lead to a more severe disease outcome in the form of a fatal heart failure. Further studies are needed to explore the role of *LDLR* and *PON1* pathways in the onset of hypercholesterolemia and atherosclerosis.

## Supplementary material

The following online material is available for this article:

Figure S1 - RFLP results of PON1 Q192R
