# Erratum: Decreased serum PON1 arylesterase activity in familial hypercholesterolemia patients with a mutated *LDLR* gene

**DOI:** 10.1590/1678-4685-GMB-2016-0287er

**Published:** 2019-05-09

In the article “Decreased serum PON1 arylesterase activity in familial hypercholesterolemia patients with a mutated *LDLR* gene”, with DOI number 10.1590/1678-4685-gmb-2016-0287, published in the jornal Genetics and Molecular Biology issue 41(3): 570-577, on page 570 the author name spelled as Abdul Rauf Siddiq should read Abdul Rauf Siddiqi.

