# Supplementary material for: Decreased serum PON1 arylesterase activity in familial hypercholesterolemia patients with a mutated LDLR gene
Source: Genet Mol Biol. 2018 Jul 23;41(3):570–7. doi: 10.1590/1678-4685-GMB-2016-0287 (PMC6136370; doi:10.1590/1678-4685-GMB-2016-0287)
Supplement: Supplementary file 1 [file 1415-4757-GMB-1678-4685-GMB-2016-0287-s001.pdf]

Supplementary Material to: “Decreased serum PON1 arylesterase activity in familial hypercholesterolemia patients with a mutated *LDLR* gene”

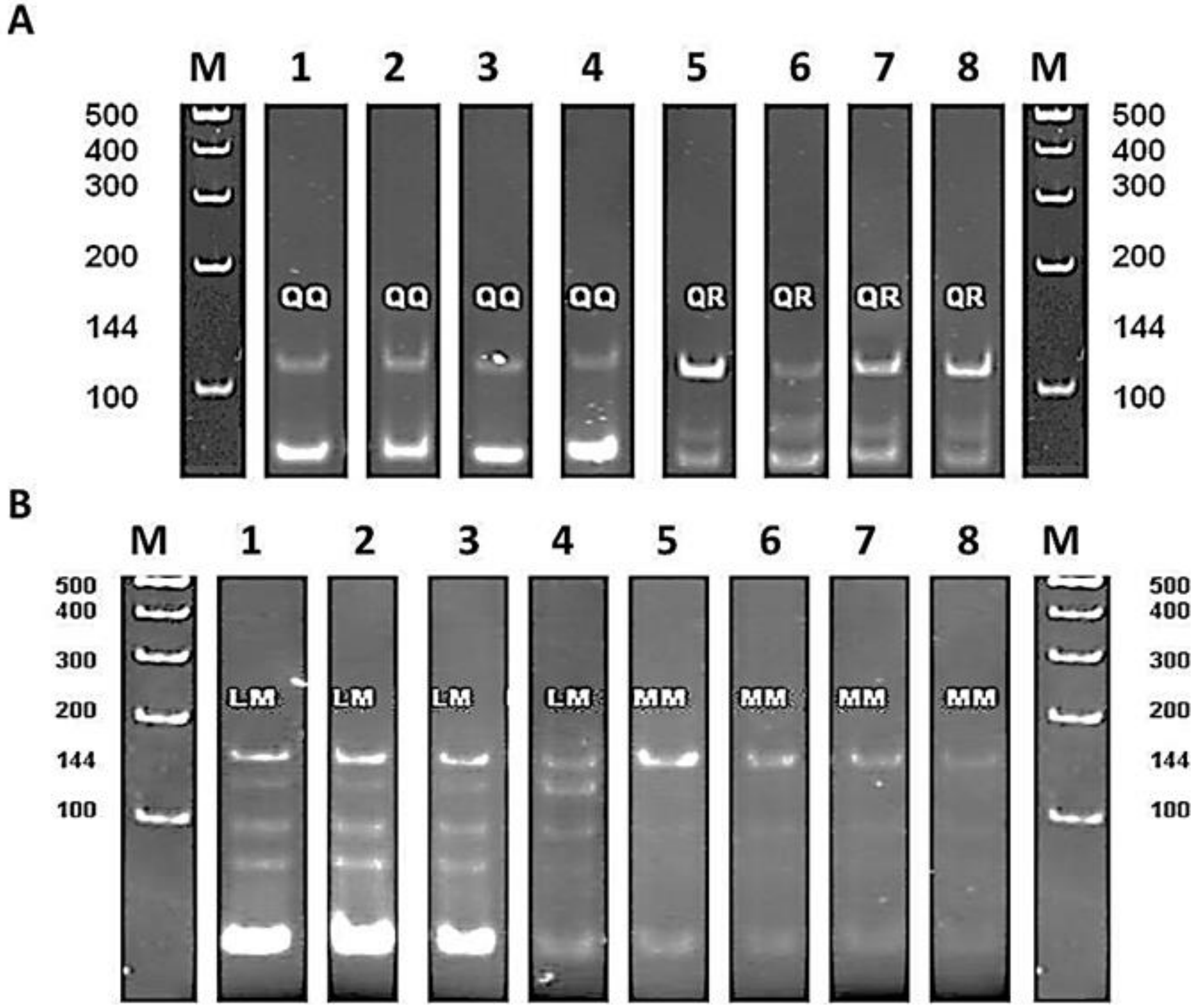

Figure S1 - RFLP results of *PON1* Q192R (Fig. **A**) and *PON1* L55M (Fig. **B**) amplified *Hinf*1 treated PCR products. M: DNA marker, Lanes 1-8: *Hinf*1 restricted PCR products
